# Fabrication of Whey Protein Isolate-Pectin Nanoparticles by Thermal Treatment: Effect of Dynamic High-Pressure Treatment

**DOI:** 10.3390/foods12234217

**Published:** 2023-11-22

**Authors:** Sohyeon Im, Owen Griffith Jones, Seung Jun Choi

**Affiliations:** 1 Department of Food Science and Biotechnology, Seoul National University of Science and Technology, Seoul 01811, Republic of Korea; sohyeon9780@gmail.com; 2 Department of Food Science, Purdue University, West Lafayette, IN 47907, USA; joneso@purdue.edu; 3 Center for Functional Biomaterials, Seoul National University of Science and Technology, Seoul 01811, Republic of Korea; 4 Research Institute of Food and Biotechnology, Seoul National University of Science and Technology, Seoul 01811, Republic of Korea

**Keywords:** complexes, dynamic high-pressure treatment, heat treatment, pectin, whey protein isolates

## Abstract

This study investigated the impact of dynamic high-pressure (DHP) treatment on the ability of whey protein isolate (WPI) to form associative complexes with pectin and to form aggregate particles after their subsequent heat treatment. Light scattering showed that DHP treatments disrupted preexisting WPI aggregates and assembled pectin chains. Complexes formed from WPI/pectin mixtures at pH 4.5 were an order of magnitude smaller when formed after DHP treatment, regardless of the degree of esterification. WPI/pectin complexes formed after DHP treatment were more stable against subsequent pH neutralization than complexes formed without DHP treatment, and WPI/high-methoxyl pectin (HMP) complexes had greater stability than WPI/low-methoxyl pectin (LMP) complexes. WPI/pectin particles prepared by thermal treatment of complexes at pH 4.5 were also smaller when prepared after DHP treatment. WPI/HMP particles were stable to subsequent pH neutralization, while WPI/LMP particles became larger after neutralization.

## 1. Introduction

Over the past few decades, biopolymer particles have attracted interest because of their potential applications as encapsulation, protection, and delivery systems in food, cosmetic, health care, and pharmaceutical products [1,2,3]. Since some of these particles have a mouthfeel that is similar to the oil droplets of some fatty foods, fat replacer is also a potential application of biopolymer particles [4]. In addition, they are useful as food structure modifiers [5] and emulsifying agents [6].

Biopolymer complexes are formed under conditions where the proteins and polysaccharides have opposite charges [7,8]. Proteins attain a net negative charge in pH conditions above the proteins’ isoelectric point (pI) and attain a net positive charge at pH conditions below their pI. Anionic polysaccharides have a negative charge above their acid dissociation constant (p*K*_a_), whereas cationic polysaccharides have a positive charge below their base dissociation constant (p*K*_b_). At pH values where proteins and polysaccharides have the same charge, the interaction between them and complexation rarely occur because of the electrostatic repulsion between them. For anionic polysaccharides, association between proteins and polysaccharides initiates at pH values just above the isoelectric pH and becomes more extensive as the pH is reduced, eventually leading to phase separation [9]. For example, critical pH values for initial complex formation and phase separation (complex coacervation) of whey protein and pectin occur at approximately pH 5.5–6 and pH 4.5, respectively, although these values are significantly influenced by the solution ionic strength and the distribution of pectin’s charge groups [10].

To improve the stability of protein/polysaccharide complexes against dissociation with changes in pH or ionic strength, complexes formed through electrostatic attraction would need further treatment [9]. Thermal treatment is one approach to induce the cross-linking of the proteins within protein/polysaccharide complexes, creating particles with gel-like internal structures [11]. When proteins are heated above their denaturation temperature, hydrophobic domains and disulfide bonds initially buried in their interiors become exposed to solvent and may interact with each other to form aggregates [12]. Therefore, the formation of irreversible protein aggregates could be possible by heating protein solutions under certain conditions. The role of associated polysaccharides during the heating of complexes is to limit protein aggregation, such that aggregates formed by heating in acidic conditions have a size distribution resembling protein aggregates formed at a relatively neutral pH [11].

Dynamic high-pressure treatment (or microfluidization) is an established homogenization technique that has been adapted as a physical treatment to promote the modification and functional properties of biological macromolecules, including proteins, starches, and non-starchy polysaccharides [13]. During dynamic high-pressure treatment (DHP treatment), very high pressure (generally up to 200 MPa) is applied to fluids containing biological macromolecules, and the pressurized fluids stream into the microchannels of the interaction chamber equipped with microfluidization devices. In the interaction chamber, fluids undergo high-velocity impact, turbulence, intense shear, and cavitation for a very short time. Although the temperature of the fluid may rise due to the high velocity in the microchannels, microfluidization is treated as a non-thermal treatment because the fluid runs through the microchannels for a very short time [14]. Although the extent of the alteration of the molecular structures of biological macromolecules varies depending on the nature of the biological macromolecules and microfluidization conditions, the molecular conformation of biological macromolecules undergoing DHP treatment may differ from that of those that do not experience DHP treatment. It suggests that DHP treatment can alter the physicochemical and functional properties of biological macromolecules. The physicochemical properties, including solubility, surface hydrophobicity, and emulsifying and foaming abilities, of ovalbumin [15], soy proteins [16], whey proteins [17], and pea proteins [18] have been successfully modified or improved by DHP treatment. Whey protein aggregates formed near pH 6 were also disrupted into smaller aggregates by DHP treatment at ~32 MPa [19]. Like food proteins, DHP treatment could induce the structural changes of food polysaccharides resulting in changes in viscosity and water and oil holding capacities [20]. DHP treatment can also disrupt small aggregates or assemblies that are naturally present within commercial samples of proteins and polysaccharides, changing their capacity to associate and their structure within formed complexes. Since relative interactivity between protein and polysaccharides is important in controlling the aggregation of heated protein-polysaccharide complexes, prior DHP treatment of proteins and polysaccharides could provide better control of the structure and potentially different physicochemical and functional properties of resulting biopolymer particles.

In this work, whey proteins were used as model protein, and pectins with high and low methoxyl contents were used as model anionic polysaccharides. Whey proteins obtained as byproducts during cheese making have various functional properties, including foaming, emulsifying, and gelling [21]. Since the impact of DHP treatment on the physicochemical properties of whey proteins was previously studied by a number of research groups [17,22], whey proteins were chosen because these reports would help in interpreting the findings in this study. The objective of this study was to investigate the impact of DHP treatment on the complexation of food proteins and polysaccharides, and the formation of protein/polysaccharide particles. The complexes of whey protein and pectin were fabricated by applying DHP treatment to individual whey protein and pectin solutions and their mixtures, after which their physicochemical properties were investigated. The physicochemical properties of the biopolymer particles fabricated from these complexes were also investigated. We hypothesized that the DHP treatment would affect the formation and physicochemical properties of the biopolymer particles fabricated by thermal treatment.

## 2. Materials and Methods

### 2.1. Materials

Whey protein isolate (9410 WPI) was kindly donated by Hilmar Ingredients (Hilmar, CA, USA). According to the manufacturer, its composition was 93.0% total protein and 2.7% ash. High-methoxyl (Classic CF 201) and low-methoxyl (Classic AB 901) pectins were donated by Herbstreith & Fox (Neuenbürg, Germany). The values of the degree of esterification (DE) for high-methoxyl and low-methoxyl pectins were 70 and 38%, respectively. Whey protein isolate (WPI), high-methoxyl pectin (HMP), and low-methoxyl pectin (LMP) were used directly from the sample containers without further purification. All other chemicals of analytical grade were purchased.

### 2.2. Sample Preparation

WPI powder (1% (*w*/*v*)) was weighed into a beaker for solubilization with distilled/deionized water containing 0.02% (*w*/*v*) sodium azide. Pectin solutions were prepared by dissolving HMP or LMP into distilled/deionized water to 0.5% (*w*/*v*). These solutions were stirred constantly at 25 °C for 8 h at 200 rpm. For the study of WPI/pectin complex formation, the prepared WPI and pectin solutions were mixed together. The mixed solution was adjusted to pH 4.5 using 0.1 and 1.0 N hydrochloric acid solutions and then stirred constantly at 25 °C for 30 min at 200 rpm. The WPI and pectin concentrations in the mixed solution were 0.5 and 0.25% (*w*/*v*), respectively. The dynamic high-pressure (DHP) treatment of the mixed solution was conducted with a microfluidizer (MN400BF, Micronox, Seongnam, Republic of Korea) with up to 3 passes at a pressure of 100 MPa at 25 °C. For the fabrication of WPI/pectin particles, the mixed solutions were heat treated for 15 min at 85 °C with weak stirring and cooled at 4 °C.

### 2.3. Particle Size and Charge Measurements

The particle sizes and surface charges of WPI/pectin particles were determined using a dynamic light scattering and micro-electrophoresis instrument (SZ-100; Horiba, Kyoto, Japan). The data for particle size are reported as the Z-average mean diameter and the data for particle surface charge are reported as the ζ-potential. All measurements were performed at 25 °C.

### 2.4. Fluorescence Spectroscopy

The surface hydrophobicity of WPI/pectin complexes was determined according to the method of Cao, Zhao, and Xiong [23]. The solution containing WPI/pectin complexes was diluted to 0.05, 0.10, 0.15, 0.20, and 0.25 mg/mL of WPI with distilled/deionized water. To 4.0 mL of the diluted solution, 20 μL of 8.0 mM 1-anilino-8-naphthalenesulfonate (ANS) (dissolved in the 100 mM phosphate buffer) was added and mixed vigorously. The fluorescence intensity was measured exactly 15 min after ANS addition using a fluorescence spectrophotometer (SpectraMax i3x; Molecular Devices, San Jose, CA, USA) with the excitation and emission wavelengths set at 390 and 470 nm, respectively. The fluorescence intensity values of the blank sample (solution containing WPI/pectin complexes without ANS) and blank reagent (distilled/deionized water without WPI/pectin complexes but with ANS) were measured and subtracted from those of samples (solution containing WPI/pectin complexes with ANS). Surface hydrophobicity was expressed as the initial slope of the fluorescence intensity versus WPI concentration plot calculated by linear regression analysis.

### 2.5. Reducing-Sugar-Ends Content Measurement

The content of reducing-sugar-ends was determined by the 3,5-dinitrosalicylic acid (DNS) method [24] with minor modification. The DNS reagent was prepared by dissolving 1 g of DNS in 50 mL of distilled water with the steady addition of 30 g of sodium potassium tartare tetrahydrate and 20 mL of 2 N NaOH until the solution’s volume totaled 100 mL with the distilled water. 2 mL of pectin solution was mixed with 1.5 mL of DNS reagent. Thereafter, the mixture was heated at 100 °C for 5 min and cooled to room temperature. The absorbance of the reaction mixture was measured at the wavelength of 540 nm using a UV/vis spectrophotometer (Optizen Pop, Mecasys, Daejeon, Republic of Korea). The calibration curve was established with galacturonic acid.

### 2.6. Statistical Analysis

All experiments were conducted in triplicate using freshly prepared samples and the data are expressed as the mean ± standard deviation. An analysis of variance (ANOVA) and Duncan’s multiple-range test (*p* ≤ 0.05) were conducted using SPSS software (ver. 26.0, IBM, Armonk, NY, USA).

## 3. Results and Discussion

### 3.1. Influence of pH on WPI/Pectin Complexes

**Figure 1:**
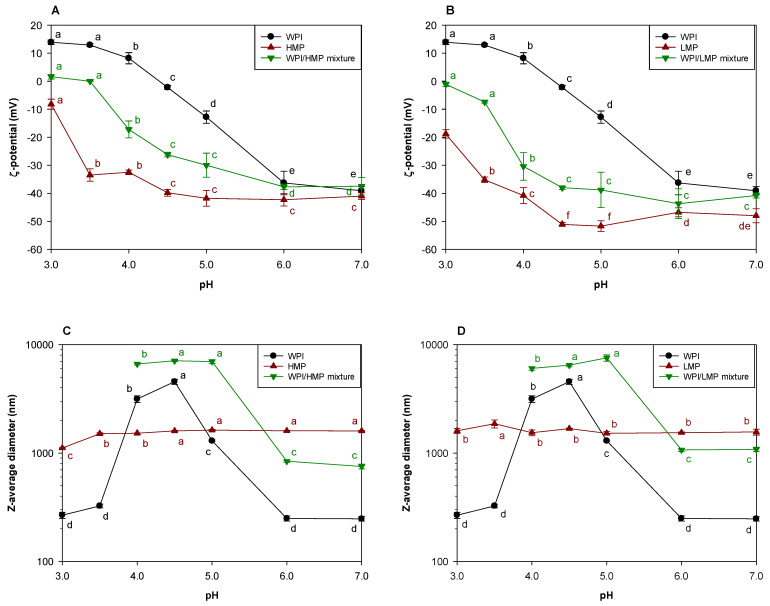
ζ-potential (**A**,**B**) and Z-average diameter (**C**,**D**) of WPI and pectin binary or mixed solutions. Solutions were prepared at the stated pH. The values denoted by the different letters indicate the significant differences within the same sample (*p* ≤ 0.05).

To assess the driving force for electrostatic interactions, the change in the ζ-potential of WPI and pectin solutions was measured as a function of pH (Figure 1A,B). WPI samples showed a switch from a negative to a positive charge near pH 4.5 which was attributed to the protein’s isoelectric point (pI). The ζ-potential values for HMP and LMP were negative over the tested pH range, and the charge magnitude of HMP was smaller than that of LMP. This difference in the ζ-potential between HMP and LMP could be due to the difference in the structural charge density between them. Decreasing pH below 4.5 dramatically decreased the charge magnitude of HMP and LMP, which can be attributed to the protonation of carboxylate-containing residues in proximity to the p*K*_a_ of pectin. Regardless of the DE of the pectins, the ζ-potential values of the WPI/pectin mixtures were intermediate and between the values of the WPI and pectin at the tested pH range indicating complex formation. Similar findings were ascribed to the charge neutralization that follows electrostatic interactions between anionic carboxyl groups on pectin and cationic amino groups on the WPI surface, with the residual negative charge above pH 4 implying an excess of the pectin chains [25].

The Z-average diameter of the WPI solution was larger than 200 nm, even at a pH far from its pI (Figure 1C,D). Since commercial WPI products are produced through a thermal process, spray-drying process, etc., the WPI in final products can be denatured and/or aggregated. Therefore, in this study, the WPI could show a relatively large Z-average diameter compared with the individual proteins (β-lactoglobulin, α-lactalbumin, and minor proteins) in WPI. At a pH (from 4 to 5) close to pI, the Z-average diameter of WPI aggregates was much larger than 1 μm because of isoelectric aggregation. The size of HMP and LMP was not affected by changes in pH, although detected sizes were much larger than expected for single pectin chains. The considerably large values of the Z-average diameter for pectins may be because some large intermolecular assemblies strongly scatter light during size measurement by dynamic light scattering [25]. In aqueous solutions, pectin molecules easily assemble through ionic, hydrophobic, and/or hydrophilic interchain interactions [26]. WPI and pectin formed large aggregates at pH values close to the pI of WPI. At these pH values, WPI/pectin complexes were formed by electrostatic attraction between cationic amino acids on the surfaces of WPI and anionic carboxyl groups on the pectin backbone. According to the previous studies [10,27], proteins bind more strongly and in greater number to polysaccharides with greater charge density than those having less charge density. When the pH of the WPI/pectin mixed solution was lower than 4, detectable precipitation occurred.

Based on the results above, since WPI and pectin had opposite charges at ≤pH 4.5, complexes of WPI and pectin can be formed through electrostatic interaction at these pH values. However, since the complexes were so large that they precipitated at <pH 4, pH 4.5 was selected for the formation of WPI/pectin complexes in subsequent experiments.

### 3.2. Influence of DHP Treatment on Individual WPI and Pectin

**Figure 2:**
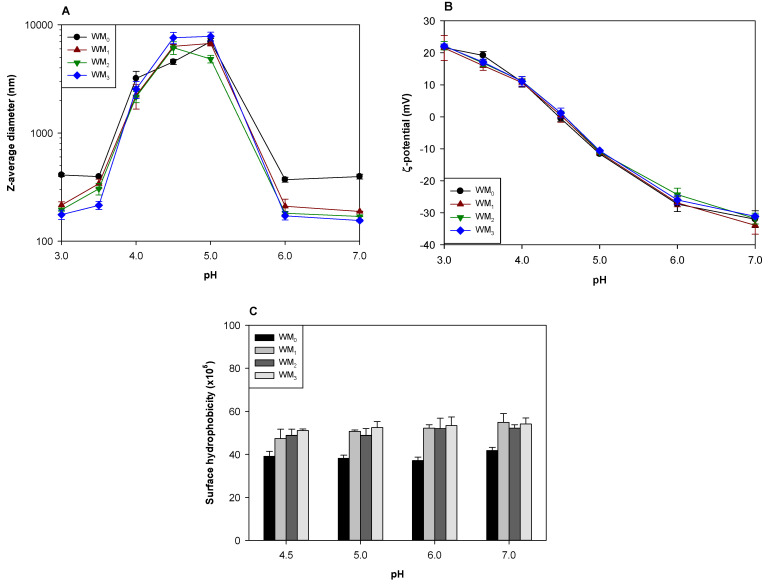
The Z-average diameter (**A**), ζ-potential (**B**), and surface hydrophobicity (**C**) of WPI after DHP treatment at pH 4.5. The pH was adjusted to final values after DHP treatment at pH 4.5. Statistical analysis is presented in Table S1.

**Figure 3:**
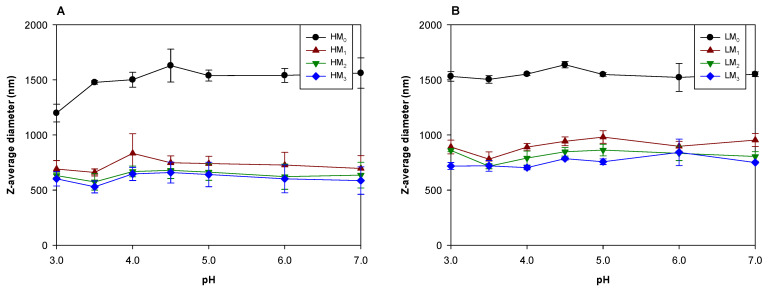
The Z-average diameter of HMP (**A**) and LMP (**B**) that underwent DHP treatment at pH 4.5. The pH was adjusted to final values after DHP treatment at pH 4.5. Statistical analysis is presented in Table S2.

DHP treatment was applied to the individual WPI and pectin solutions that were already adjusted to pH 4.5, and the resulting samples were characterized by their size, surface charge, and hydrophobicity (Figure 2 and Figure 3). The WPI solution subjected to x passes through a microfluidizer at pH 4.5 is labeled as WM_x_. The HMP solution subjected to x passes through a microfluidizer at pH 4.5 is labeled as HM_x_ and the LMP solution subjected to x passes through a microfluidizer at pH 4.5 is labeled as LM_x_.

Figure 2A shows that DHP treatment had little initial impact on protein at pH 4.5, as the size of WM_1–3_ was slightly larger than WM_0_. However, WM_1–3_ samples behaved differently than WM_0_ after subsequent pH adjustments. WPI samples adjusted to pH 4.5 but with no DHP treatment (WM_0_) remained as micrometer-sized aggregates after adjustment to pH 4 or 5 and reduced to less than 500 nm with further acidification or neutralization (Figure 2A). Since changing pH from 4.5 to 3 or 7 increased electrostatic repulsion between WPI, the large WPI aggregates formed at pH 4.5 dissociated into smaller aggregates. The size of WM_0_ measured after adjusting pH to 7 (or 3) was about twice the size of WPI originally solubilized in pH 7 (or 3) (Figure 1C and Figure 2A), indicating that electrostatic repulsions at pH 7 or 3 failed to fully dissociate WPI aggregates formed at pH 4.5 into the original WPI aggregates. Instead, hydrophobic interactions between WPI aggregates may have maintained assembly. The size of WM_1–3_ was similar to WM_0_ between pH 4 and 5 but, after adjusting pH to 7 or 3, became much smaller than that of WPI originally solubilized at pH 7 or 3. It indicated that the tremendous forces of shear, impact, and cavitation during DHP treatment disrupted the stronger interactions among WPI aggregates so that subsequent pH changes allowed them to dissociate into smaller aggregates [28]. DHP treatments were previously shown to reduce the size of WPI aggregates and increase dispersibility in water [16,19,22].

There was no difference in the ζ-potential between WM_x_ at a given pH (Figure 2B), indicating that their net-charge was almost the same. However, since the charged amino acids buried inside WPI aggregates can be exposed through the disassociation of WPI aggregates during DHP treatment, the charge density of WM_1–3_ may be greater than that of WM_0_.

Adjusting pH to <4 dramatically increased the surface hydrophobicity of WM_x_ (Figure S1). However, it would not indicate that decreasing pH caused the exposure of the hydrophobic domains originally buried in the interior of WPI aggregates. The surface hydrophobicity determined using ANS is generally overestimated at acidic pH than at neutral pH because anionic ANS can interact with the amino acids having positively charged residues on the protein surface [29]. As shown in Figure 2C, WM_1–3_ showed greater surface hydrophobicity than WM_0_ at pH 4.5, but increasing the number of DHP treatments did not further increase their surface hydrophobicity. As noted above, since the tremendous forces during DHP treatment dissociated the original WPI aggregates, the hydrophobic domains buried into WPI aggregates would be exposed to their surface, resulting in an increase in surface hydrophobicity. Also, the increased surface hydrophobicity after DHP treatment could be due to the exposure of the cationic amino acids originally buried in the interior of WPI aggregates during DHP treatment. Since the structure of WPI aggregates was loosened due to the electrostatic repulsion at a pH higher than pI and the hydrophobic domains existing inside were exposed, the surface hydrophobicity of WM_x_ slightly increased with pH.

As shown in Figure 3, DHP treatment reduced the size of pectins, regardless of their DE. DHP treatment also reduced the width of particle size distributions (not shown). Pectin can easily form aggregates through ionic, hydrophobic, and/or hydrophilic interchain interactions in an aqueous solution [26]. The breakdown of interchain interactions between pectin molecules may be one of the possible reasons for the size reduction of pectins after DHP treatment. The breakdown of glycosidic linkages between galacturonic acids in pectins may also be one of the possible reasons for this observation [30]. Since the galacturonic acids of pectin link with each other through glycosidic linkages, the content of the reducing-sugar-ends would increase when the glycosidic linkages of the pectin are broken down. The values of the reducing-sugar-ends content of HMP and LMP were 0.049 ± 0.001 and 0.025 ± 0.000 mM, respectively, and these values were rarely changed by DHP treatment. It indicated that the change in pectin size due to DHP treatment may mainly be due to the dissolution of pectin aggregates rather than the breakdown of the glycosidic linkages in pectins. According to a previous report [31], the high hydrostatic pressure treatment (200–600 MPa for 30 min) did not cause a significant change in the reducing sugar content of apple pectin. Although DHP treatment was employed in this study while the previous experiment used a high hydrostatic pressure treatment making a direct application to this study challenging, the unchanged reducing sugar content after DHP treatment in this study was likely attributed to the lower pressure and much shorter processing time compared to the previous experiment with the high hydrostatic pressure treatment. This is also consistent with study on the microfluidization of xanthan gum, which disrupted the formation of large fibrous xanthan gum assemblies with no evidence of primary chain hydrolysis [32].

### 3.3. Influence of DHP Treatment on WPI/Pectin Complexes

Before the fabrication of WPI/pectin nanoparticles by heat-setting, the pH of WPI/pectin solutions was adjusted to 4.5 before applying DHP treatment. WPI/pectin complexes subjected to x passes through a microfluidizer at pH 4.5 are labeled as WHM_x_C and WLM_x_C for WPI/HMP and WPI/LMP complexes, respectively.

**Figure 4:**
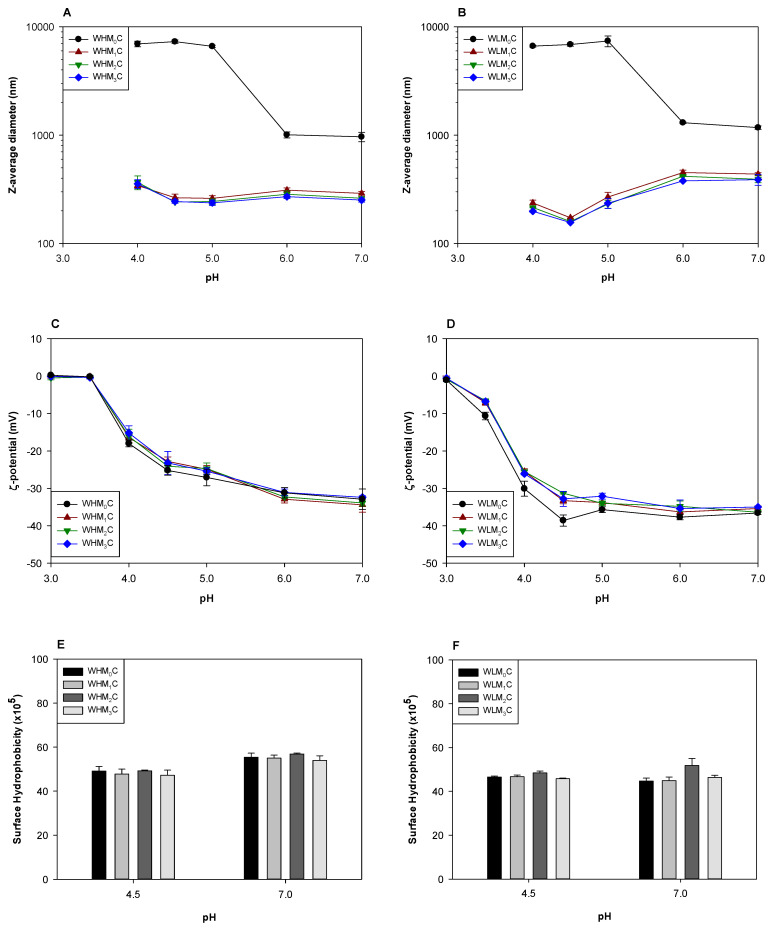
The Z-average diameter (**A**,**B**), ζ-potential (**C**,**D**), and surface hydrophobicity (**E**,**F**) of WPI/pectin complexes after DHP treatment at pH 4.5. The pH was adjusted to final values after complexation at pH 4.5. Statistical analysis is presented in Tables S3 and S4.

Among samples that did not undergo DHP treatment, large WPI aggregates originating from the commercial sample participated in complexation with pectin in WHM_0_C and WLM_0_C through electrostatic interaction at pH 4.5. Large objects observed by scattering were likely due to these large aggregates forming complexes with pectin (Figure 4A,B). Adjusting the pH of the WPI/pectin complex solution to 4 or 5 had little effect on the size of the WPI/pectin complexes formed at pH 4.5. There would be sufficient electrostatic attraction to stabilize the WPI/pectin complexes formed at pH 4.5 because pH 4 and 5 are very close to the pI of WPI. When the pH was increased to ≥6, the size of WHM_0_C and WLM_0_C decreased. There were two possible explanations for the size reduction of the WPI/pectin complexes. First, the decrease in the electrostatic attraction and the concurrent increase in the electrostatic repulsion between WPI and pectin molecules could dissociate WPI/pectin complexes into the individual WPI and pectin molecules. Second, the increase in electrostatic repulsion may cause the dissociation of the large-size complexes into small-size ones.

At pH 4.5, the sizes of WHM_1–3_C and WLM_1–3_C were much smaller than that of WHM_0_C and WLM_0_C, but increasing the number of passes with DHP treatment had no further effect on the size of WPI/pectin complexes (Figure 4A,B). This smaller complex size was because WPI aggregates and pectins were reduced in size by DHP treatments so complexes could be assembled on a smaller scale. Smaller pectin chains are better able to interact with the smaller WPI produced after DHP treatments, further preventing the formation of large assemblies near the protein’s pI. This observation was consistent with a study that showed DHP treatments of xanthan gum facilitated the formation of smaller complexes with whey protein because of the disruption of large polysaccharide assemblies [32].

Since pH values above WPI’s pI increase the electrostatic repulsion between WPI and pectin, it was expected that adjusting the pH from 4.5 to 7 would decrease the size of WHM_1–3_C, as it did for WHM_0_C. However, increasing the pH from 4.5 to 7 had little effect on the size of WHM_1–3_C (Figure 4A). As described above, WHM_1–3_C were formed by interaction between WPI aggregates and HMP, both of which were reduced in size by DHP treatment. While the dominant interaction was between the charge groups of WPI and HMP, it is plausible that hydrophobic and hydrogen-bond interactions between the newly exposed surfaces of WPI aggregates and HMP chains played a significant role in the formation of WHM_1–3_C. HMP is known to form gels through hydrogen bonds between nonionized carboxyl groups and hydrophobic interactions between methoxyl groups [33]. Such groups could interact with relatively hydrophobic regions of WPI exposed after DHP treatment, as described by Figure 2C. Evidence of significant contributions for hydrogen bonding has been previously found in β-lactoglobulin complexes with HMP [34]. Although increased pH would promote the dissociation of WPI and HMP, these hydrophobic and hydrogen-bond interactions could hold WPI aggregates and HMP together over a wider pH range.

The surface charge and hydrophobicity of WHM_1–3_C changed significantly with subsequent pH adjustments with no noticeable effects of DHP treatments (Figure 4C,E). This indicated that the surface structure of WHM_1–3_C was very similar to that of WHM_0_C. pH increasing in increments from 4.5 to 7 increased the magnitude of the ζ-potential magnitude of WHM_1–3_C by about 10 mV and their surface hydrophobicity by about 7 × 10^5^. The increase in the anionic surface charge could be attributed to the deprotonation of the cationic amino acids of WPI aggregates on the surface that were not involved in interactions with HMP. Such a change in the surface charge by incremental increases in pH may lead to a change in the surface structure of WHM_1–3_C, and a change in surface hydrophobicity due to the pH increasing in increments indicated that a change in the surface structure occurred. However, since the size of WHM_1–3_C did not change significantly with increasing pH, it was expected that the change in their surface structure did not coincide with changes to the internal structure of WHM_1–3_C. A plausible explanation for the increase in surface hydrophobicity is the dissociation of some HMP from the surface of the complexes as the pH was increased.

Unlike WHM_1–3_C, the size of WLM_1–3_C increased with increased pH values (Figure 4B). Also, the ζ-potential and surface hydrophobicity did not change (Figure 4D,F), indicating that an excess of the anionic groups of LMP dominated the surface charge of WLM_1–3_C. Since the DE of LMP is considerably lower than that of HMP, hydrophobic and hydrogen-bond interactions between WPI aggregates and LMP were expected to be less important and would not prevent some dissociation of LMP from the surface of WLM_1–3_C. When pH increased from 4.5, electrostatic repulsions between the anionic groups of WPI and LMP could then allow some chain segments (e.g., ends) of LMP to partially detach from the surface and be stretched into the aqueous phase, resulting in a transformation from a smooth spherical structure to a ‘hairy’ (or corona-like) structure. Such a transformation can explain the relatively constant surface charge despite the increased size of WLM_1–3_C as the pH increased.

### 3.4. Influence of DHP Treatment on WPI/Pectin Particles

To examine the impact of DHP treatment on the properties of WPI/pectin particles fabricated by heat treatment, WPI and pectin were treated with DHP and mixed to create complexes (WHM_x_C and WLM_x_C) before heat treatment to create the particles. WPI/pectin particles fabricated with WHM_x_C and WLM_x_C are labeled as WHM_x_P and WLM_x_P, respectively.

**Figure 5:**
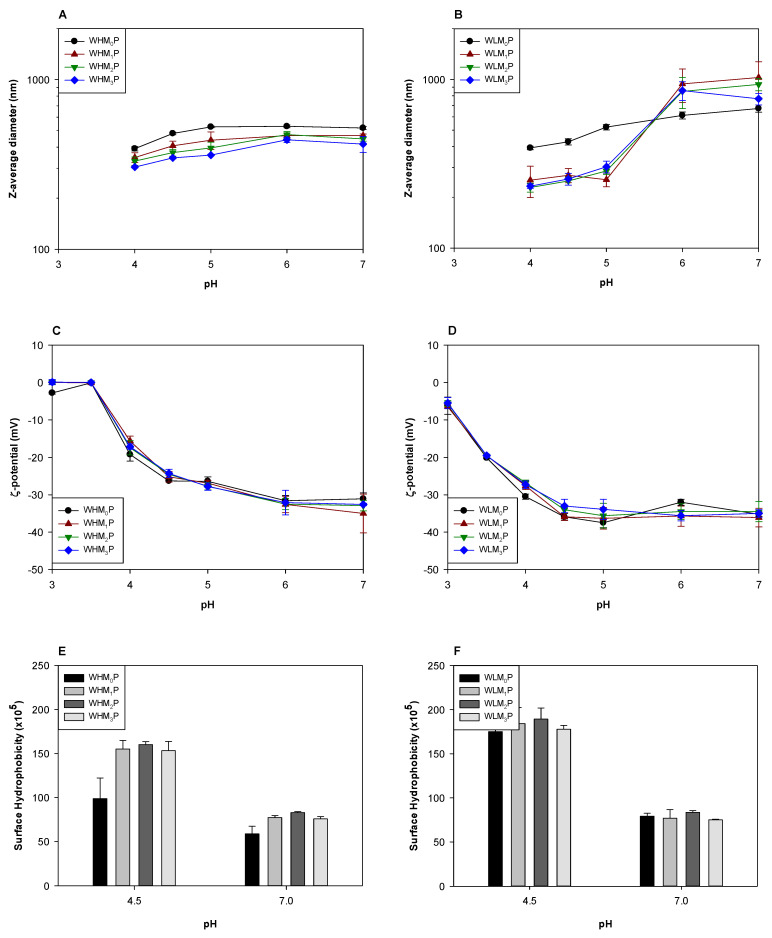
The Z-average diameter (**A**,**B**), ζ-potential (**C**,**D**), and surface hydrophobicity (**E**,**F**) of the particles fabricated by heating WPI/pectin complexes that were previously treated by DHP at pH 4.5. The pH was adjusted to final values after heat treatment at pH 4.5. Statistical analysis is presented in Tables S5 and S6.

Heat treatment at pH 4.5 dramatically reduced the sizes of the complexes that were prepared without DHP so that WHM_0_P and WLM_0_P were smaller than WHM_0_C (Figure 4A and Figure 5A) and WLM_0_C (Figure 4B and Figure 5B). The surface charges of the particles were not significantly affected by thermal treatment or DHP (Figure 5C,D). The surface hydrophobicity values were increased by heat treatment at pH 4.5 for all samples (Figure 4E and Figure 5E).

The size reduction of WHM_0_C and WLM_0_P after heat treatment can be explained by the significant structural rearrangement of the complexes to form aggregate particles, as observed in prior studies [35]. During heat treatment, the WPI molecules in the complexes unfold (or denature) and the electrostatic interaction between WPI and pectin may be weakened. As a result, the denatured WPI released from the complexes formed new WPI aggregates by electrostatic and hydrophobic interactions [36], and the newly exposed hydrophobic groups could explain the increased surface hydrophobicity of all samples after heat treatment. During cooling, pectins electrostatically interact with the cationic patches on the surface of WPI aggregates and limit the extent of WPI aggregation [37]. Therefore, the regenerated particles with a shell layer consisting of pectin are stable suspensions due to the anionic surface charge [38].

WHM_1–3_P and WLM_1–3_P were smaller than WHM_0_P and WLM_0_P (Figure 4A and Figure 5A), which could be attributed to the smaller sizes of complexes formed after the DHP treatment. Unlike WHM_0_P and WLM_0_P, WHM_1–3_P and WLM_1–3_P were slightly larger at pH 4.5 than the complexes (WHM_1–3_C and WHM_1–3_C) from which they were formed. Such an increase in size could again be attributed to the partial dissociation of the pectins followed by aggregation processes to generate aggregates in a preferred size range. The aggregation of WPI tends to prefer certain aggregate sizes under specific pH, ionic strength, and concentration conditions [39,40] as well as at relative interactions with oppositely charged polysaccharides [41]. Complexes made from DHP-treated samples increased in perceived size with heat treatment because they were smaller than the preferred size, unlike the very large complexes formed without DHP treatment. However, pectins were apparently more effective in restricting aggregation after DHP treatments, possibly because the interactions between WPI and pectins in WHM_1–3_C and WLM_1–3_C were greater in extent than in WHM_0_C or WLM_0_C.

Increasing pH increased the size of WHM_x_P by about 100 nm, independent of the number of passes with DHP treatment (Figure 5A). A strong surface charge indicated electrostatic repulsion between particles in the pH range from 4.5 to 7 (Figure 5C), so size increases were unlikely to be due to aggregation between particles. Instead, size increases could be due to the loosened structure of WHM_x_P due to the increased electrostatic repulsion between WPI and HMP, forming a diffuse layer on the particle surface. The increase in the pH also led to a decrease in surface hydrophobicity of WHM_x_P rather than an increase (Figure 5E), which confirmed that pectin did not detach from the particles at pH 7.

Interestingly, increasing pH to ≥6 dramatically increased the size of WLM_1–3_P (Figure 5B). Since the surface charge was consistent across the pH range and with unheated complexes (Figure 5D), LMP was likely to maintain consistent coverage on the particle surface. As described above, LMP is less capable of hydrogen-bond and hydrophobic interactions with WPI when compared to HMP, so LMP could be detached to a greater degree from the particle surface. This detachment could result in full dissociation followed by some particle–particle aggregation or detachment of most of the LMP chains to create large diffuse layers around the particle surface. Since the carboxyl groups of LMP could be strongly bound with the cationic amino acid residues of WPI, it is likely that at least some of the LMP remained attached and created ‘hairy’ regions on the particles. As with HMP, surface hydrophobicity decreased with increased pH (Figure 5F) indicating either that the LMP was still covering a significant portion of the particle surface or that the aggregation of destabilized particles secluded the newly exposed hydrophobic regions.

## 4. Conclusions

The study demonstrated that DHP treatment impacted the supramolecular structures of WPI and pectin, leading to an improved capacity to form associative complexes and their heat-set particles. DHP treatment of WPI and pectin decreased their size in aqueous suspension and led to formation of complexes with smaller sizes, regardless of the DE of the pectins participating in the complexation. Without DHP treatment, micrometer-sized complexes at pH 4.5 dissociated significantly as the pH increased back to 7. Among the samples treated with DHP, some dissociation of WPI/LMP complexes occurred with a pH increase, while the apparent dissociation of WPI/HMP complexes was negligible and indicated that hydrogen-bonding and/or hydrophobic interactions had a role in complex formation. The particles fabricated by the thermal treatment of the complexes all possessed diameters in the range of 200–500 nm, with particles prepared with DHP treatments being much smaller. The sizes of WPI/HMP particles were more stable against subsequent pH changes when compared to WPI/LMP particles, with the relative stability largely unaffected by DHP treatment. However, the initial smaller size of particles formed after DHP treatments led to smaller WPI/HMP particles over the pH range of 4–7 and smaller WPI/LMP particles over the pH range of 4–5. Therefore, these findings suggested that DHP treatment could be a useful tool to prepare protein/carbohydrate complexes and their heat-set particles, particularly when using commercially sourced protein isolates and polysaccharides that have a significant content of aggregates and assemblies. Also, since protein/carbohydrate complexes prepared through DHP treatment and their particles, particularly WPI/HMP complexes and their particles, had a relatively high stability against pH changes, they appear to have a high potential for application as food materials including Pickering emulsion stabilizers and fat replacers.

## Data Availability

The data used to support the findings of this study can be made available by the corresponding author upon request.

## Supplementary Materials

The following supporting information can be downloaded at: https://www.mdpi.com/article/10.3390/foods12234217/s1, Figure S1: Impact of pH adjustment on the surface hydrophobicity of WPI undergone DHP treatment at pH 4.5. The pH was adjusted to final values after DHP treatment at pH 4.5; Table S1: Statistical analysis of Z-average diameter, ζ-potential, and surface hydrophobicity of WPI after DHP treatment at pH 4.5; Table S2: Statistical analysis of Z-average diameter of HMP and LMP undergone DHP treatment at pH 4.5; Table S3: Statistical analysis of Z-average diameter, ζ-potential, and surface hydrophobicity of WPI/HMP complexes after DHP treatment at pH 4.5; Table S4: Statistical analysis of Z-average diameter, ζ-potential, and surface hydrophobicity of WPI/LMP complexes after DHP treatment at pH 4.5; Table S5: Statistical analysis of Z-average diameter, ζ-potential, and surface hydrophobicity of particles fabricated by heating WPI/HMP complexes that were previously treated by DHP treatment at pH 4.5; Table S6: Statistical analysis of Z-average diameter, ζ-potential, and surface hydrophobicity of particles fabricated by heating WPI/LMP complexes that were previously treated by DHP treatment at pH 4.5.
