# Supplementary material for: Fabrication of Whey Protein Isolate-Pectin Nanoparticles by Thermal Treatment: Effect of Dynamic High-Pressure Treatment
Source: Foods. 2023 Nov 22;12(23):4217. doi: 10.3390/foods12234217 (PMC10705890; doi:10.3390/foods12234217)
Supplement: Supplementary file 1 [file foods-12-04217-s001.zip › foods-2700030-supplementary.pdf]

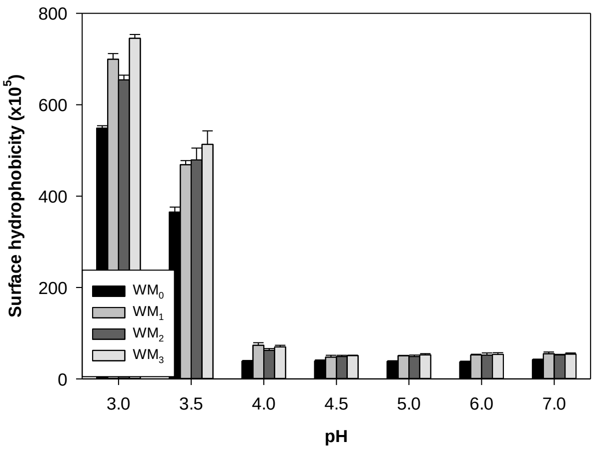

Supplementary Figure S1. Impact of pH adjustment on the surface hydrophobicity of WPI undergone DHP treatment at pH 4.5. The pH was adjusted to final values after DHP treatment at pH 4.5.

Supplementary Table S1. Statistical analysis of Z-average diameter, ζ-potential, and surface hydrophobicity of WPI after DHP treatment at pH 4.5.

Z-average diameter (nm)							
Sample	pH						
	3	3.5	4	4.5	5	6	7
WM_0	^{A}409.0 ± 13.9^{d}	^{A}394.9 ± 6.2^{d}	^{A}3198.4 ± 516.2^{c}	^{C}4549.2 ± 249.3^{b}	^{AB}6985.6 ± 483.6^{a}	^{A}369.3 ± 19.2^{d}	^{A}395.5 ± 20.5^{d}
WM_1	^{B}215.1 ± 15.3^{c}	^{B}339.4 ± 34.6^{c}	^{B}2228.4 ± 568.0^{b}	^{B}6326.0 ± 238.8^{a}	^{B}6730.8 ± 366.5^{a}	^{B}210.3 ± 34.0^{c}	^{B}188.0 ± 1.0^{c}
WM_2	^{BC}194.7 ± 6.0^{d}	^{B}304.5 ± 37.3^{d}	^{B}2161.2 ± 256.0^{c}	^{B}6141.6 ± 826.2^{a}	^{C}4826.5 ± 391.9^{b}	^{B}181.1 ± 5.1^{d}	^{BC}168.9 ± 0.5^{d}
WM_3	^{C}175.6 ± 17.6^{c}	^{C}214.0 ± 18.5^{c}	^{AB}2538.4 ± 455.1^{b}	^{A}7592.7 ± 913.0^{a}	^{A}7826.1 ± 722.5^{a}	^{B}171.2 ± 14.7^{c}	^{C}154.8 ± 4.1^{c}
ζ-potential (mV)							
Sample	pH						
	3	3.5	4	4.5	5	6	7
WM_0	^{A}21.6 ± 0.7^{a}	^{A}19.2 ± 1.2^{a}	^{A}10.9 ± 1.6^{b}	^{A}-0.5 ± 0.9^{c}	^{B}-11.6 ± 0.4^{d}	^{A}-27.3 ± 2.3^{e}	^{A}-32.0 ± 2.6^{f}
WM_1	^{A}21.5 ± 3.9^{a}	^{B}16.0 ± 1.5^{b}	^{A}10.7 ± 1.2^{c}	^{A}0.5 ± 2.2^{d}	^{AB}-11.2 ± 0.5^{e}	^{A}-26.9 ± 1.2^{f}	^{A}-33.9 ± 2.8^{g}
WM_2	^{A}22.2 ± 1.3^{a}	^{B}16.8 ± 1.1^{b}	^{A}11.2 ± 0.1^{c}	^{A}1.2 ± 0.1^{d}	^{AB}-11.0 ± 0.4^{e}	^{A}-24.3 ± 2.0^{f}	^{A}-31.8 ± 1.7^{g}
WM_3	^{A}22.1 ± 0.6^{a}	^{AB}17.1 ± 0.9^{b}	^{A}11.1 ± 1.5^{c}	^{A}1.2 ± 1.5^{d}	^{A}-10.6 ± 0.3^{e}	^{A}-26.0 ± 1.5^{f}	^{A}-31.1 ± 0.5^{g}
Surface hydrophobicity ($\times 10^5$)							
Sample	pH						
	-	-	-	4.5	5	6	7
WM_0				^{B}39.1 ± 2.3^{ab}	^{B}38.2 ± 1.5^{b}	^{B}37.1 ± 1.6^{b}	^{B}41.8 ± 1.5^{a}
WM_1				^{A}47.4 ± 4.2^{b}	^{A}50.7 ± 0.7^{ab}	^{A}52.2 ± 1.6^{ab}	^{A}54.9 ± 4.0^{a}
WM_2				^{A}48.9 ± 2.9^{a}	^{A}48.4 ± 3.2^{a}	^{A}52.0 ± 4.8^{a}	^{A}52.2 ± 1.5^{a}
WM_3				^{A}51.0 ± 0.8^{a}	^{A}52.5 ± 2.7^{a}	^{A}53.3 ± 4.0^{a}	^{A}54.1 ± 2.8^{a}

The pH was adjusted to final values after DHP treatment at pH 4.5.

The values with different capital-letter superscripts in a same column are significantly different by Duncan's multiple-range test ($p \leq 0.05$).

The values with different small-letter superscripts in a same row are significantly different by Duncan's multiple-range test ($p \leq 0.05$).

Supplementary Table S2. Statistical analysis of Z-average diameter of HMP and LMP undergone DHP treatment at pH 4.5.

Z-average diameter (nm)							
Sample	pH						
	3	3.5	4	4.5	5	6	7
HM_0	$^{A}1199.2 \pm 80.7$	$^{A}1477.7 \pm 16.4^{a}$	$^{A}1501.6 \pm 67.7^{a}$	$^{A}1629.7 \pm 149.4^{a}$	$^{A}1539.6 \pm 50.0^{a}$	$^{A}1540.9 \pm 63.1^{a}$	$^{A}1562.4 \pm 137.8^{a}$
HM_1	$^{B}691.2 \pm 76.8^{a}$	$^{B}660.0 \pm 31.9^{a}$	$^{B}832.9 \pm 178.9^{a}$	$^{B}747.6 \pm 61.6^{a}$	$^{B}741.1 \pm 65.5^{a}$	$^{B}726.6 \pm 115.8^{a}$	$^{B}696.4 \pm 116.3^{a}$
HM_2	$^{B}630.8 \pm 26.0^{a}$	$^{BC}574.5 \pm 74.4^{a}$	$^{B}668.9 \pm 48.9^{a}$	$^{B}679.6 \pm 75.0^{a}$	$^{B}662.9 \pm 74.4^{a}$	$^{B}621.6 \pm 114.6^{a}$	$^{B}635.8 \pm 117.1^{a}$
HM_3	$^{B}601.9 \pm 65.2^{a}$	$^{C}529.0 \pm 54.6^{a}$	$^{B}646.7 \pm 59.9^{a}$	$^{B}659.4 \pm 95.1^{a}$	$^{B}641.1 \pm 111.0^{a}$	$^{B}602.3 \pm 126.6^{a}$	$^{B}586.1 \pm 124.9^{a}$

Sample	pH						
	3	3.5	4	4.5	5	6	7
HM_0	$^{A}1532.2 \pm 43.6^{b}$	$^{A}1504.8 \pm 35.1^{b}$	$^{A}1553.1 \pm 14.4^{ab}$	$^{A}1638.0 \pm 28.9^{a}$	$^{A}1549.3 \pm 16.5^{ab}$	$^{A}1522.5 \pm 126.0^{b}$	$^{A}1551.5 \pm 22.2^{ab}$
HM_1	$^{B}890.8 \pm 62.4^{a}$	$^{B}780.3 \pm 66.0^{b}$	$^{B}889.9 \pm 32.1^{a}$	$^{B}943.5 \pm 39.8^{a}$	$^{B}981.2 \pm 57.5^{a}$	$^{B}897.8 \pm 44.6^{a}$	$^{B}954.3 \pm 60.0^{a}$
HM_2	$^{B}860.8 \pm 13.1^{a}$	$^{B}716.4 \pm 22.9^{b}$	$^{C}790.3 \pm 63.0^{ab}$	$^{C}847.4 \pm 38.5^{a}$	$^{C}862.8 \pm 51.9^{a}$	$^{B}833.2 \pm 65.5^{a}$	$^{C}805.1 \pm 43.5^{a}$
HM_3	$^{C}718.6 \pm 31.3^{b}$	$^{B}719.8 \pm 48.8^{b}$	$^{D}703.5 \pm 19.0^{b}$	$^{D}785.7 \pm 12.1^{ab}$	$^{D}757.4 \pm 26.1^{ab}$	$^{B}842.5 \pm 120.9^{a}$	$^{C}750.0 \pm 5.8^{ab}$

The pH was adjusted to final values after DHP treatment at pH 4.5.

The values with different capital-letter superscripts in a same column are significantly different by Duncan's multiple-range test ($p \leq 0.05$).

The values with different small-letter superscripts in a same row are significantly different by Duncan's multiple-range test ($p \leq 0.05$).

Supplementary Table S3. Statistical analysis of Z-average diameter, ζ-potential, and surface hydrophobicity of WPI/HMP complexes after DHP treatment at pH 4.5.

Z-average diameter (nm)							
Sample	pH						
	-	-	4	4.5	5	6	7
WHM$_0$C			^{A}6955.1 ± 409.4^{ab}	^{A}7308.8 ± 270.4^{a}	^{A}6607.1 ± 175.1^{b}	^{A}1009.4 ± 64.2^{c}	^{A}966.5 ± 95.5^{c}
WHM$_1$C			^{B}343.1 ± 16.3^{a}	^{B}264.5 ± 21.3^{c}	^{B}261.1 ± 15.5^{c}	^{B}310.3 ± 16.0^{a}	^{B}290.2 ± 12.1^{bc}
WHM$_2$C			^{B}366.5 ± 53.1^{a}	^{B}241.3 ± 6.1^{b}	^{B}244.0 ± 14.6^{b}	^{B}284.5 ± 21.2^{b}	^{B}261.6 ± 8.6^{b}
WHM$_3$C			^{B}355.0 ± 33.1^{a}	^{B}243.7 ± 8.9^{b}	^{B}237.3 ± 13.7^{b}	^{B}270.7 ± 10.9^{b}	^{B}251.6 ± 10.9^{b}
ζ-potential (mV)							
Sample	pH						
	3	3.5	4	4.5	5	6	7
WHM$_0$C	^{A}0.2 ± 0.2^{a}	^{A}-0.2 ± 0.1^{a}	^{A}-18.0 ± 0.9^{b}	^{A}-25.2 ± 1.3^{c}	^{A}-27.1 ± 2.2^{c}	^{A}-31.2 ± 1.4^{d}	^{A}-32.9 ± 2.7^{d}
WHM$_1$C	^{AB}-0.1 ± 0.1^{a}	^{A}-0.2 ± 0.3^{a}	^{A}-16.4 ± 1.3^{b}	^{A}-22.8 ± 1.2^{c}	^{A}-24.9 ± 1.0^{d}	^{A}-32.9 ± 1.0^{e}	^{A}-34.4 ± 2.0^{e}
WHM$_2$C	^{B}-0.5 ± 0.5^{a}	^{A}-0.3 ± 0.4^{a}	^{A}-16.1 ± 1.9^{b}	^{A}-24.1 ± 1.5^{c}	^{A}-24.7 ± 1.5^{c}	^{A}-32.3 ± 0.4^{d}	^{A}-33.9 ± 0.8^{d}
WHM$_3$C	^{AB}-0.1 ± 0.1^{a}	^{A}-0.4 ± 0.4^{a}	^{A}-15.2 ± 1.9^{b}	^{A}-23.2 ± 3.1^{c}	^{A}-25.4 ± 0.9^{c}	^{A}-31.1 ± 0.9^{d}	^{A}-32.4 ± 0.4^{d}
Surface hydrophobicity (×10^{5})							
Sample	pH						
	-	-	-	4.5	-	-	7
WHM$_0$C				^{A}49.1 ± 2.1^{b}			^{A}55.4 ± 1.9^{a}
WHM$_1$C				^{A}47.8 ± 2.2^{b}			^{A}55.0 ± 1.4^{a}
WHM$_2$C				^{A}49.2 ± 0.4^{b}			^{A}56.9 ± 0.4^{a}
WHM$_3$C				^{A}47.3 ± 2.3^{b}			^{A}54.0 ± 2.1^{a}

The pH was adjusted to final values after DHP treatment at pH 4.5.

The values with different capital-letter superscripts in a same column are significantly different by Duncan's multiple-range test ($p \leq 0.05$).

The values with different small-letter superscripts in a same row are significantly different by Duncan's multiple-range test ($p \leq 0.05$).

Supplementary Table S4. Statistical analysis of Z-average diameter, ζ-potential, and surface hydrophobicity of WPI/LMP complexes after DHP treatment at pH 4.5.

Z-average diameter (nm)							
Sample	pH						
	-	-	4	4.5	5	6	7
WLM_0C			$^{A}6634.2 \pm 140.8^{b}$	^{A} 866.5 ± 235.0^{ab}	$^{A}7399.5 \pm 851.5^{a}$	$^{A}1305.6 \pm 21.8^{c}$	$^{A}1175.6 \pm 41.0^{c}$
WLM_1C			$^{B}236.6 \pm 14.7^{b}$	$^{B}173.0 \pm 2.6^{c}$	$^{B}268.1 \pm 28.6^{b}$	$^{B}451.5 \pm 23.1^{a}$	$^{B}436.8 \pm 14.1^{a}$
WLM_2C			$^{B}215.2 \pm 1.9^{c}$	$^{B}159.6 \pm 1.0^{d}$	$^{B}230.9 \pm 19.3^{c}$	$^{C}417.2 \pm 3.1^{a}$	$^{C}291.8 \pm 27.8^{b}$
WLM_3C			$^{B}198.2 \pm 4.6^{b}$	$^{B}156.1 \pm 1.3^{c}$	$^{B}235.2 \pm 25.4^{b}$	$^{D}378.2 \pm 3.5^{a}$	$^{B}388.3 \pm 44.3^{a}$
ζ-potential (mV)							
Sample	pH						
	3	3.5	4	4.5	5	6	7
WLM_0C	$^{A}-1.1 \pm 0.4^{a}$	$^{B}-10.7 \pm 1.0^{b}$	$^{B}-30.1 \pm 2.0^{c}$	$^{B}-38.6 \pm 1.5^{e}$	$^{B}-35.7 \pm 0.8^{d}$	$^{B}-37.7 \pm 0.7^{de}$	$^{B}-36.6 \pm 0.4^{de}$
WLM_1C	$^{A}-0.6 \pm 0.6^{a}$	$^{A}-7.1 \pm 0.7^{b}$	$^{A}-25.5 \pm 0.7^{c}$	$^{A}-33.3 \pm 0.8^{d}$	$^{AB}-33.8 \pm 1.7^{d}$	$^{AB}-36.3 \pm 0.5^{e}$	$^{A}-35.4 \pm 0.7^{e}$
WLM_2C	$^{A}-0.9 \pm 0.1^{a}$	$^{A}-6.6 \pm 0.2^{b}$	$^{A}-25.5 \pm 0.3^{c}$	$^{A}-31.3 \pm 0.2^{d}$	$^{AB}-34.0 \pm 0.6^{e}$	$^{A}-34.8 \pm 1.4^{e}$	$^{B}-36.4 \pm 0.1^{f}$
WLM_3C	$^{A}-0.6 \pm 0.1^{a}$	$^{A}-6.8 \pm 0.3^{b}$	$^{A}-26.1 \pm 0.3^{c}$	$^{A}-32.8 \pm 2.0^{d}$	$^{A}-32.1 \pm 0.8^{d}$	$^{AB}-35.4 \pm 2.3^{e}$	$^{A}-35.0 \pm 0.1^{e}$
Surface hydrophobicity ($\times 10^5$)							
Sample	pH						
	-	-	-	4.5	-	-	7
WLM_0C				$^{A}46.6 \pm 0.4^{a}$			$^{B}44.7 \pm 1.3^{a}$
WLM_1C				$^{B}46.7 \pm 0.8^{a}$			$^{B}44.9 \pm 1.6^{a}$
WLM_2C				$^{A}48.4 \pm 0.9^{a}$			$^{A}51.8 \pm 3.3^{a}$
WLM_3C				$^{B}45.8 \pm 0.3^{a}$			$^{B}46.4 \pm 1.0^{a}$

The pH was adjusted to final values after DHP treatment at pH 4.5.

The values with different capital-letter superscripts in a same column are significantly different by Duncan's multiple-range test ($p \leq 0.05$).

The values with different small-letter superscripts in a same row are significantly different by Duncan's multiple-range test ($p \leq 0.05$).

Supplementary Table S5. Statistical analysis of Z-average diameter, ζ-potential, and surface hydrophobicity of particles fabricated by heating WPI/HMP complexes that were previously treated by DHP treatment at pH 4.5.

Z-average diameter (nm)							
Sample	pH						
	-	-	4	4.5	5	6	7
WHM_0P			^{A}391.5 ± 12.3^{c}	^{A}481.6 ± 8.4^{b}	^{A}526.7 ± 6.9^{a}	^{A}530.7 ± 9.4^{a}	^{A}519.1 ± 10.4^{a}
WHM_1P			^{B}347.2 ± 22.8^{c}	^{B}407.5 ± 26.6^{b}	^{B}439.6 ± 52.6^{ab}	^{B}468.1 ± 16.3^{a}	^{B}468.0 ± 10.2^{a}
WHM_2P			^{BC}330.9 ± 18.8^{c}	^{C}371.0 ± 17.5^{b}	^{BC}395.8 ± 32.7^{b}	^{B}473.9 ± 21.1^{a}	^{B}447.1 ± 13.5^{a}
WHM_3P			^{C}305.0 ± 3.8^{c}	^{C}345.2 ± 3.7^{b}	^{C}358.9 ± 0.9^{b}	^{B}441.9 ± 16.4^{a}	^{C}416.9 ± 44.4^{a}
ζ-potential (mV)							
Sample	pH						
	3	3.5	4	4.5	5	6	7
WHM_0P	^{B}-2.8 ±0.3^{b}	^{A}-0.1 ± 0.1^{a}	^{B}-19.2 ± 1.8^{c}	^{B}-26.3 ± 0.3^{d}	^{A}-26.4 ± 1.2^{d}	^{A}-31.6 ± 1.4^{e}	^{A}-31.1 ± 1.6^{e}
WHM_1P	^{A}0.0 ± 0.1^{a}	^{A}-0.1 ± 0.2^{a}	^{A}-15.6 ± 1.3^{b}	^{AB}-25.2 ± 1.4^{c}	^{A}-26.9 ± 0.7^{c}	^{A}-32.6 ± 2.2^{d}	^{A}-35.0 ± 5.2^{d}
WHM_2P	^{A}0.1 ± 0.5^{a}	^{A}0.0 ± 0.2^{a}	^{AB}-17.4 ± 1.9^{b}	^{A}-24.5 ± 0.3^{c}	^{A}-27.7 ± 1.0^{d}	^{A}-32.5 ± 1.5^{e}	^{A}-32.9 ± 1.4^{e}
WHM_3P	^{A}0.1 ± 0.7^{a}	^{A}0.0 ± 0.1^{a}	^{AB}-17.1 ± 0.7^{b}	^{A}-24.3 ± 1.1^{c}	^{A}-27.8 ± 1.0^{d}	^{A}-32.1 ± 3.3^{e}	^{A}-32.6 ± 0.3^{e}
Surface hydrophobicity (×10^5)							
Sample	pH						
	-	-	-	4.5	-	-	7
WHM_0P				^{B}98.8 ±23.5^{a}			^{B}58.8 ± 8.7^{a}
WHM_1P				^{A}155.1 ± 9.8^{a}			^{A}77.3 ± 2.2^{b}
WHM_2P				^{A}160.2 ± 3.2^{a}			^{A}82.9 ± 1.1^{b}
WHM_3P				^{A}153.3 ± 10.4^{a}			^{A}75.8 ± 2.4^{b}

The pH was adjusted to final values after heat treatment at pH 4.5.

The values with different capital-letter superscripts in a same column are significantly different by Duncan's multiple-range test ($p \leq 0.05$).

The values with different small-letter superscripts in a same row are significantly different by Duncan's multiple-range test ($p \leq 0.05$).

Supplementary Table S6. Statistical analysis of Z-average diameter, ζ-potential, and surface hydrophobicity of particles fabricated by heating WPI/LMP complexes that were previously treated by DHP treatment at pH 4.5.

Z-average diameter (nm)							
Sample	pH						
	-	-	4	4.5	5	6	7
WLM_0P			$^{A}392.8 \pm 9.3^{d}$	$^{A}427.1 \pm 18.3^{d}$	$^{A}521.7 \pm 19.2^{c}$	$^{B}612.4 \pm 29.1^{b}$	$^{B}673.0 \pm 33.6^{a}$
WLM_1P			$^{B}252.7 \pm 53.3^{b}$	$^{B}269.9 \pm 26.2^{b}$	$^{C}254.5 \pm 23.8^{b}$	$^{A}941.5 \pm 212.1^{a}$	$^{A}1027.1 \pm 248.1^{a}$
WLM_2P			$^{B}228.7 \pm 14.0^{b}$	$^{B}250.0 \pm 13.9^{b}$	$^{BC}286.3 \pm 12.8^{b}$	$^{AB}850.2 \pm 178.8^{a}$	$^{AB}935.7 \pm 78.7^{a}$
WLM_3P			$^{B}232.9 \pm 7.3^{b}$	$^{B}256.6 \pm 20.7^{b}$	$^{B}303.3 \pm 24.2^{b}$	$^{AB}859.8 \pm 108.8^{a}$	$^{AB}767.4 \pm 60.7^{a}$
ζ-potential (mV)							
Sample	pH						
	3	3.5	4	4.5	5	6	7
WLM_0P	$^{A}-6.2 \pm 2.3^{a}$	$^{B}-20.1 \pm 0.3^{b}$	$^{B}-30.5 \pm 0.7^{c}$	$^{B}-35.9 \pm 0.9^{de}$	$^{A}-37.5 \pm 1.3^{e}$	$^{A}-32.0 \pm 0.8^{c}$	$^{A}-35.2 \pm 0.6^{d}$
WLM_1P	$^{A}-6.5 \pm 0.2^{a}$	$^{A}-19.5 \pm 0.0^{b}$	$^{A}-27.6 \pm 1.0^{c}$	$^{B}-35.9 \pm 1.0^{d}$	$^{A}-36.3 \pm 2.9^{d}$	$^{A}-35.7 \pm 2.7^{d}$	$^{A}-36.1 \pm 2.5^{d}$
WLM_2P	$^{A}-5.6 \pm 0.8^{a}$	$^{A}-19.6 \pm 0.3^{b}$	$^{A}-26.8 \pm 0.7^{c}$	$^{AB}-34.0 \pm 0.8^{d}$	$^{A}-35.6 \pm 3.3^{d}$	$^{A}-34.5 \pm 2.1^{d}$	$^{A}-34.5 \pm 2.7^{d}$
WLM_3P	$^{A}-5.5 \pm 1.5^{a}$	$^{A}-19.5 \pm 0.3^{b}$	$^{A}-27.3 \pm 1.0^{c}$	$^{A}-33.0 \pm 1.8^{d}$	$^{A}-33.9 \pm 2.7^{d}$	$^{A}-35.6 \pm 1.4^{d}$	$^{A}-35.0 \pm 1.0^{d}$
Surface hydrophobicity ($\times 10^5$)							
Sample	pH						
	-	-	-	4.5	-	-	7
WLM_0P				$^{A}175.0 \pm 8.4^{a}$			$^{AB}79.2 \pm 3.4^{b}$
WLM_1P				$^{A}184.2 \pm 18.3^{a}$			$^{AB}76.9 \pm 9.7^{b}$
WLM_2P				$^{A}189.3 \pm 12.5^{a}$			$^{A}85.6 \pm 1.9^{b}$
WLM_3P				$^{A}177.9 \pm 4.2^{a}$			$^{B}75.0 \pm 0.8^{b}$

The pH was adjusted to final values after heat treatment at pH 4.5.

The values with different capital-letter superscripts in a same column are significantly different by Duncan's multiple-range test ($p \leq 0.05$).

The values with different small-letter superscripts in a same row are significantly different by Duncan's multiple-range test ($p \leq 0.05$).
